# Effects of central hypovolemia induced by tilt table on the Doppler- based renal resistive index in healthy volunteers

**DOI:** 10.1186/cc13337

**Published:** 2014-03-17

**Authors:** A Sommese, A Lima, J Van Bommel, J Bakker

**Affiliations:** 1 Erasmus MC, Rotterdam, the Netherlands

## Introduction

The renal resistive index (RI) determined by Doppler ultrasonography allows a semiquantitative evaluation of kidney vasculature at the bedside. Interpretation of the RI in clinical practice is difficult due to interaction with cardiac output, heart rate (HR) and blood pressure [1],[2]. The impact of global hemodynamics on the RI remains to be evaluated. This study aims to investigate the relationship between the RI and changes in central hemodynamic during a central hypovolemia model in healthy volunteers (HV).

## Methods

Eleven healthy volunteers (27 ± 8 years; eight male) participated in this study. Two different models were performed: the first model was performed by applying the head-up tilt (HUT) test. The complete maneuver was done by a 10-minute step that consisted of tilting the table from a supine position (Sup) to an angle of 70° (HUT) and back to supine (Sup'). The second model was performed by applying three consecutive valsalva maneuvers. Global hemodynamics included stroke volume (SV), HR, and mean arterial pressure, which were continuously measured non-invasively with a Finometer. At least three RI readings were obtained and averaged from the right and left kidneys in all HV.

## Results

**Figure 1:**
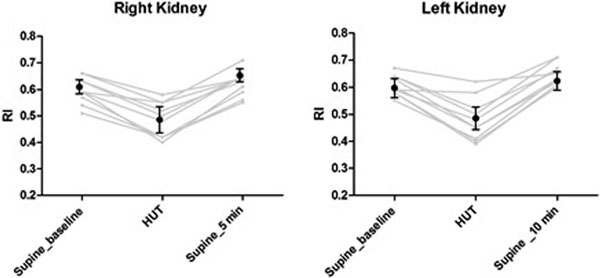
**Doppler-based renal resistive index in both kidneys during HUT**.

All HV had a significant decrease of SV from 83 ± 17 ml to 63 ± 14 ml and an increase in HR from 67 ± 10 bpm to 88 ± 13 bpm during the HUT. Figure 1 shows the temporal changes of mean RI in both kidneys. A significant decrease in the RI in both kidneys was seen during HUT. After the move back to supine, RI returned to baseline values, with a significant variation of RI in the early measurements on the right kidney compared with late measurements on the left kidney (0.67 ± 0.05 vs. 0.61 ± 0.05, *P *< 0.05). Valsalva maneuvers significantly increased the RI in the right and left kidneys, from 0.6 ± 0.04 and 0. 6.± 0.05 to 0.7 ± 0.1 and 0.68 ± 0.15 (*P *< 0.05), respectively.

## Conclusion

These preliminary results showed that Doppler renal RI was affected equally in both kidneys during HUT, suggesting an effect of hemodynamic alterations during our model of central hypovolemia.
